# Can participative leadership and LMX congruence promote collective organizational engagement?

**DOI:** 10.1371/journal.pone.0346702

**Published:** 2026-04-10

**Authors:** Mozhi Li, Jinying Cao, Qiheng Sun

**Affiliations:** 1 School of Economics and Management, Ningxia Institute of Science and Technology, Shizuishan, Ningxia, China; 2 School of Economics and Management, Bangkok Thonburi University, Bangkok, Thailand; 3 Business School, Qinghai Institute of Technology, Xining, Qinghai, China; Woldia University, ETHIOPIA

## Abstract

Employee engagement has been widely recognized as an important indicator of organizational functioning and performance. While prior research has primarily examined engagement at the individual level, emerging studies suggest that engagement may spread through social interactions, leading to a shared, collective form of organizational engagement. Building on social contagion theory, this study examines the relationships between participative leadership, leader–member exchange (LMX) congruence (i.e., the degree of agreement between leaders’ and employees’ LMX ratings), and collective organizational engagement. Using matched survey data from 243 respondents nested within 54 commercial organizations operating in mainland China across manufacturing, information technology, services, real estate, and fast-moving consumer goods sectors, we employed polynomial regression and response surface analysis to examine patterns of congruence and incongruence in LMX ratings. The results indicate that participative leadership is positively associated with collective organizational engagement. When leaders’ and employees’ LMX ratings are congruent, the High–High profile is associated with higher levels of collective organizational engagement than the Low–Low profile. Under incongruent conditions, the Low–High profile is associated with higher collective organizational engagement than the High–Low profile. Furthermore, the interaction between participative leadership and specific LMX congruence profiles shows differential predictive relationships with collective organizational engagement. These findings contribute to the literature by extending engagement research to the collective level and by clarifying how leadership practices and leader–employee perceptual alignment relate to collective organizational engagement.

## Introduction

In the context of rapid organizational transformation driven by artificial intelligence (AI), AI enhances organizational performance by optimizing processes and reshaping the interaction patterns between leaders and subordinates [1]. Leaders and their subordinates represent critical human capital within organizations, and their work-related attitudes and motivational states (such as vigor and dedication) form the foundation for enterprises to achieve excellence [2]. Kahn (1990) defined engagement as individuals’ willingness to devote themselves physically, cognitively, and emotionally to their work [3]. Building on Kahn’s foundational work, subsequent scholars conceptualized engagement as a sustained, a persistent and active psychological state characterized by vigor, dedication, and absorption with three dimensions: vigor, dedication, and concentration [4]. As a core predictor of corporate performance [2,5], engagement has become increasingly prominent in organizational behavior research and managerial practice. Prior research suggests that organizations characterized by higher levels of engagement tend to demonstrate stronger performance outcomes [5,6].

Existing studies on employee engagement mainly adopt an individual perspective. Some studies take employee engagement as a dependent variable, analyzing the influence of factors such as employment relationship climate, voice behavior, and leadership styles on employee engagement [6,7], while other studies use employee engagement as an independent variable, investigating its influence on job performance [1,2,8]. However, engagement is not just an individual-level phenomenon, but also a team-level one [9–11]. Enterprise practitioners note that organizational engagement is perhaps a factor influencing corporate performance. In this context, some scholars propose concepts such as team engagement [11] and collective organizational engagement [2]. Unlike employee engagement, collective organizational engagement (or team engagement) emphasizes a shared perception (consensus) among team members of actively participating in work, highlighting a fusion of collective emotions, cognition, and motivation, so it involves an emergent state where the whole is greater than the sum of its parts [9,11]. Adopting Barrick et al.’s perspective on collective organizational engagement, this research defines collective organizational engagement as a shared perception of a team’s psychological, cognitive, and emotional involvement in their work [2^]^. In the field of organizational management, collective organizational engagement is a new notion, so analyzing its antecedents will unveil its role as a “black box” in enhancing corporate performance.

Leaders play a vital role in human resources of an organization and also act as the agents of the organization. Therefore, leaders are a key factor in studying collective organizational engagement. Participative leadership, as a leadership style, originates from the distributed leadership theory, the shared leadership theory, and the empowering leadership theory [12]. It refers to a leadership style of leaders encouraging subordinates to take on greater job responsibility [13]. Older generations of Chinese entrepreneurs often favor centralized leadership, but increasing evidence proves that newer generations of entrepreneurs are adopting participative leadership. Participative leadership focuses on motivating instead of controlling employees so that employees can achieve individual and collective goals through self-management. For example, large multinational corporations such as Google and Apple have benefited from participative leadership, proving that participative leadership can enhance team cooperation [14]. As participative leadership allows employees to participate in decision-making, encourages team members to jointly set goals and take action [15], and fosters an atmosphere of joint decision-making between the leader and the employees, it can boost employees’ enthusiasm for sharing knowledge and ideas, achieve shared authority and responsibility, and enhance team efficiency [14]. Moreover, participative leaders can set themselves as examples to motivate their subordinates to work hard for creating a culture where leaders encourage subordinates and subordinates motivate each other, thus enhancing overall work engagement. Therefore, studying the influence of participative leadership on collective organizational engagement can deepen the integration research of leadership styles and collective organizational engagement.

From the perspective of guanxi (relationships) in traditional Chinese sociocultural contexts, it is worth discussing which leader-employee relationships can maximize employees’ enthusiasm. Leader-member exchange (LMX) reveals the quality of the leader-employee exchange relationship. LMX is a key variable of dyadic interaction [16]. Role Theory suggests that the building and development of LMX is a role-making process involving a series of events [16]. Leaders transmit information on their expectations for employees’ roles to employees and adjust the leader-employee relationship based on employees’ responses. Employees who meet expectations will develop high-quality, trust-based exchanges with their leaders, while those who do not meet expectations will face low-quality, transactional exchanges. Incongruence between employees’ and leaders’ perceptions often occurs in the transmission and reception of role information [17], particularly when roles are ambiguous or undefined. Employees and leaders will interpret their roles according to their own understanding, leading to divergent interpretations, which will hinder the employees’ work engagement and, consequently, dampen collective organizational engagement. Existing research often uses either the leader’s or employees’ LMX ratings to represent their perceptions of LMX quality, but this approach has recently come into question [18]. Many studies have found cognitive differences between leaders’ and subordinates’ perceptions of the leader-subordinate relationship [19,20]. Therefore, it is essential to study the impact of both congruent and incongruent LMX perceptions on collective organizational engagement to deepen LMX research and expand the research on collective organizational engagement antecedents.

In conclusion, leadership and LMX congruence are two critical factors affecting collective organizational engagement. However, few studies have analyzed their interaction association on work outcomes. From a practical standpoint, the association of leadership and LMX on organizational outcomes are inseparable, so the interaction between leadership styles and LMX deserves more attention. This research investigates the association of participative leadership, LMX congruence, and their interaction on collective organizational engagement, with the objectives to explain the causes of the low engagement level and help leaders adjust leader-subordinate relationships to win the trust and loyalty of employees and enhance collective organizational engagement, thus increasing the corporate value and offering new insights to engagement research.

Building on intrinsic motivation theory, role theory, and social contagion theory, this study proposes an integrated mechanism to explain how participative leadership and LMX congruence jointly influence collective organizational engagement. Participative leadership enhances employees’ intrinsic motivation by satisfying their needs for autonomy, competence, and relatedness. Simultaneously, LMX congruence reflects the degree to which leaders and employees share aligned role expectations. When role expectations are aligned, psychological consistency reduces ambiguity and strengthens reciprocal exchanges. Through social contagion processes, motivated and psychologically aligned employees transmit positive engagement states to other members, resulting in collective organizational engagement as an emergent team-level construct.

## 1. Theoretical foundation and hypotheses development

### 1.1 Participative leadership and collective organizational engagement

Participative leadership, a leadership style, refers to the practice of leaders encouraging their subordinates to take on more responsibilities at work [13]. Such leaders generally employ incentives such as encouragement, support, and influence to encourage employees to actively participate in decision-making. Participative leaders often consult his subordinates on issues such as decision-making and strive for consensus among their team members [21]. Existing studies primarily analyze the association of participative leadership on organizational engagement [12], employees’ job performance [21,22], and organizational citizenship behavior [23], but rarely explore its association on collective organizational engagement. Unlike individual engagement, which reflects an individual’s personal psychological investment in work, collective organizational engagement represents a shared, emergent team-level state characterized by collective vigor, dedication, and absorption. It is not simply the arithmetic aggregation of individual engagement scores; rather, it emerges through interaction, shared experiences, and emotional contagion processes among team members. Following the referent-shift consensus model, collective organizational engagement reflects a shared perception that “we as a team” are engaged in our work [9]. It is not merely the sum of individual engagement but an emergent state of an organization arising from social interaction. This research believes that participative leadership, as a critical contextual factor, elicits collective organizational engagement.

According to the intrinsic motivation theory, when the work environment can satisfy individuals’ demands for autonomy, competence, and relationships, employees will get motivated by the inherent enjoyment and meaningfulness of work itself [24,25]. Participative leadership grants employees the right to participate in decision-making and problem-solving, which enhances employees’ sense of control and autonomy over their work and satisfies their need for autonomy [15]. Meanwhile, resource, information, and additional support from leaders can raise employees’ self-efficacy, that is, fulfilling their competence needs [26]. This process can change employees’ extrinsic work motivation into intrinsic one, so that employees show high levels of individual engagement—characterized by great vigor, dedication, and absorption in work. Moreover, participative leadership can create a unified organizational atmosphere, strengthen employees’ sense of organizational support, and enhance their recognition of their team [27]. Team members with a strong sense of team identity will develop a greater collective sense [28], so they will prioritize team needs and thus make psychological inputs, such as loyalty and dedication.

Furthermore, the social contagion theory suggests that subordinates can imitate their leaders’ emotions and behaviors [29] and also inspire others in the same way [30]. Therefore, as participative leadership actively encourages employee involvement in decision-making, subordinates will imitate this behavior to encourage other people [31,32], thus gradually fostering an atmosphere within the organization where people encourage each other to participate in organizational activities. Due to social contagion, employees gradually develop a shared belief in active participation in organizational activities. In other words, employees within the organization will show a stronger sense of organizational belonging, higher enthusiasm, and greater collective organizational engagement.

Based on the above analysis, the following hypothesis is developed.

*H1:* Participative leadership is positively associated with collective organizational engagement.

### 1.2 LMX Profiles and Collective Organizational Engagement

LMX levels can be rated as high and low. A “high LMX rating” means that leaders or employees perceive their exchanges as socio-emotional, while a “low LMX rating” indicates that leaders or employees perceive their exchanges as transactional [20]. According to the dyadic nature of LMX and cognitive differences between leaders (L) and employees (E), four LMX profiles are classified based on their ratings: ① High (L) – High (E) (congruence), ② Low (L) – Low (E) (congruence), ③ High (L) – Low (E) (incongruence), ④ Low (L) – High (E) (incongruence). The first two profiles represent LMX congruence, while the latter two represent LMX incongruence. Table 1 summarizes the four LMX profiles.

**Table 1:** LMX ratings of leaders and employees in 2*2 matrix.

Leaders’ perceptions of LMX	Employees’ perceptions of LMX	Socio-emotional roles/transactional roles	Matching results
High	High	Leaders and subordinates are congruent in their ratings of socioemotional roles, expectations, behaviors, and resource exchange.	Congruence in Socio-emotional roles
Low	Low	Leaders and subordinates are congruent in their ratings of transactional roles, expectations, behaviors, and resource exchanges.	Congruence in social transactional rules
High	Low	Leaders and subordinates are incongruent in their ratings of roles, expectations, behaviors, and resource exchanges.	Incongruence
Low	High	Leaders and subordinates are incongruent in their ratings of roles, expectations, behaviors, and resource exchanges.	Incongruence

**Note:** This table was compiled based on the research of Matta, Scott, and Koopman (2015).

According to Role Theory (Dansereau, 1975), social roles of individuals and interactions with these roles (including their social positions, interactions with others, and societal expectations for the positions) can shape individuals’ behaviors [33]. The building and development of LMX refer to a role-making process involving a series of events [16]. Leaders transmit information about their role expectations for employees and adjust the leader-employee relationship based on employees’ responses.

In cases of LMX congruence, leaders and employees have matched role expectations, congruent in role expectations. It can help understand the other party’s intention and improve job satisfaction and engagement [20]. (1) When both leaders and employees rate their relationship as high, leaders, guided by affective cognition, often use non-material incentives (such providing care and fostering a family-like corporate culture) [20^]^. Upon perceiving the leader’s emotional inputs, employees will reciprocate by taking the leader as a respected teacher and role model [34] and following the norm of reciprocity to take extra-role tasks and enhance work engagement [35]. (2) When both leaders and employees rate their relationship as low, leaders’ material incentives align with employees’ expectations [36]. In line with the principle of mutual benefit [37], employees will work diligently for more rewards. According to the social contagion theory, such proactive behaviors will spread among employees through imitation [38], thus improving collective organizational engagement. Therefore, this paper hypothesizes that under the combined effect of Role Theory and Social Contagion theory, both “Low(L)-Low(E)” and “High(L)-High(E)” profiles have a positive effect on collective organizational engagement.

Unlike the “Low (L) – Low (E)” profile that merely values the exchange of benefits, the “High (L) – High (E)” profile can foster emotional attachment with employees to motivate them to over-fulfill job tasks and offer selfless mutual assistance. Constrained by management costs, effective “non-material incentives” tend to have a greater effect than material incentives [39]. Therefore, this study hypothesizes that “High(L)-High(E)” congruence has a greater positive effect on collective organizational engagement than"Low(L)-Low(E).

#### Based on the above analysis, the following hypotheses are proposed

*H2a:* The “High(L)-High(E)” profile is positively associated with collective organizational engagement.

*H2b:* The “Low(L)-Low(E)” profile is positively associated with collective organizational engagement.

*H2c:* The “High(L)-High(E)” profile has a greater positive effect on collective organizational engagement than “Low(L)-Low(E).”

LMX incongruence refers to the cognitive differences between leaders and subordinates regarding their relationships. It mainly consists of two profiles: “High (L)-Low (E)” and “Low (L)-High (E).” LMX rating incongruence will provoke psychological conflicts, expectation gaps, and negative emotions between leaders and subordinates [40]. These expectation gaps and negative emotions may affect employees’ vigor, dedication, and concentration at work. Existing studies primarily focus on the topic of “which party—employee or leader—has a greater influence in their exchange” and mainly adopt the following two perspectives. Employees are the primary decision-makers of their role behavior. Leaders possess more work resources [20]. From one perspective, scholars argue that employees, as role recipients, interpret and respond to role expectations based on their own perceptions [41]. As employees are the main decision-makers of their own behavior, their self-perception is a critical factor affecting their work attitudes and behaviors. Therefore, employees are more likely to behave according to their LMX perceptions [3]. Employees’ perceptions of LMX have a greater impact on employee engagement and organizational citizenship behavior [42]. From the other perspective, scholars believe that leaders have organizational authority, so they can control the allocation of critical work resources [43]. They can allocate work resources based on their LMX perceptions, preferentially assigning scarce and valuable resources to employees with good leader-member relationships so that these employees can better complete job tasks [44]. Therefore, the effect of LMX incongruence on collective organizational engagement may vary by the LMX profile.

For the “Low (L)-High (E)” profile, the first perspective (taking the employee as the decision-maker) suggests that Employees take their leaders as respected teachers [34], whereas leaders take employees merely as work collaborators providing necessary job support [20]. Despite this discrepancy in their role perceptions, employees, influenced by their high LMX rating, will respond positively to strengthen the leader-employee relationship. For the “Low (L)-High (E)” profile, the second perspective (leaders controlling work resources) indicates that leaders rate the LMX low, so they will not provide any additional benefits or tasks to employees [20]. Leaders will just offer basic resource support to employees, so that employees can get more discretionary time and energy after completing their job tasks [3], and thus foster positive emotions and attitudes toward leaders, thereby enhancing work engagement. In conclusion, both perspectives suggest that the “Low (L)-High (E)” profile has a positive effect on collective organizational engagement.

For the “High (L)-Low (E)” profile, the first perspective (taking employees as the primary decision-makers) argues that employees see leaders as a task supervisor [45], while leaders take employees as trusted assistants and assign them excessive tasks beyond their role expectations. In this case, a mismatch occurs between the role information of employees and leaders. Consequently, employees will take on excessive tasks and develop a sense of exploitation, which breeds resentment and misunderstanding, thus preventing employees from positively evaluating their leaders, fulfilling leaders’ expectations and engaging actively in work. For the “High (L)-Low (E)” profile, the second perspective (leaders controlling work resources) suggests that employees expect role-bound tasks, but leaders leverage their authority to assign excessive work resources and tasks [20], which consumes extra time and energy of employees. Leaders allocate work resources based on their high LMX ratings, which will cause stress for low-LMX employees, thus further hindering employees’ understanding of the leaders’ intentions, preventing effective communication with the leaders, and diminishing the cutlivation of employee engagement. Existing studies prove that subordinates’ LMX perceptions has a greater effect on their work attitudes than leaders’ LMX perceptions [46]. Therefore, this paper argues that in the LMX incongruence, the “High (L) -Low (E)” profile leads to lower collective organizational engagement.

Based on the above analysis, the following hypotheses are developed.

*H3:* The effect of LMX incongruence on collective organizational engagement vary by profile.

*H3a:* The “Low (L)-High (E)” profile is positively associated with collective organizational engagement.

*H3b:* The “High (L)-Low (E)” profile is negatively associated with collective organizational engagement.

*H3c:* The “Low (L)-High (E)” profile shows a significantly stronger positive association with collective organizational engagement than the “High (L)-Low (E)” profile.

### 1.3 Interaction between LMX Congruent Profiles and Participative Leadership

The participative leader sets an example and delegates authority to convey expectations to subordinates, breaks down collective goals into several sub-goals, motivates the engagement of subordinates in organizational decision-making, sets personal goals, and helps subordinates align their personal objectives with collective goals, thus fostering self-management, facilitating team cooperation, and strengthening team cohesion [15]. In the “High (L)-High (E)” LMX profile, employees and leaders can establish high-quality relationships through interactions, so they will build trust and maintain long-lasting, high-quality relationships [47]. Employees can perceive trust from their leader in interactions and thus willingly uphold the leader’s authority, put positive emotions in their work, and enhance their engagement. Therefore, the interaction between participative leadership and the “High (L)-High (E)” profile has a positive effect on collective organizational engagement.

According to the analysis of H2, the “Low (L)-Low (E)” profile has a positive effect on collective organizational engagement, but the leader-subordinate relationship in this profile is more transactional, relying on extrinsic motivation. When participative leadership encourages employees to actively engage in organizational decision-making and activities, it will positively affect employee engagement. In this sense, the interaction between participative leadership and “Low (L)-Low (E)” profile also positively affects collective organization engagement. However, from the perspective of the intrinsic motivation theory, the interaction between participative leadership and the “High (L)-High (E)” profile may have the greatest positive effect on collective organizational engagement.

*H4:* The interaction between participative leadership and LMX congruent profiles is positively associated with collective organizational engagement.

*H4a:* The interaction between participative leadership and the “High (L)-High (E)” profile is positively associated with collective organizational engagement.

*H4b:* The interaction between participative leadership and the “Low (L)-Low (E)” profile is positively associated with collective organizational engagement.

According to *H3*, employees’ LMX perceptions play a decisive role in shaping their behaviors [42,46]. In the “Low (L)-High (E)” profile, participative leadership can provide employees with more intrinsic motivation, raising employees’ enthusiasm and enhancing their work engagement. Therefore, the interaction between participative leadership and the “Low (L)-High (E)” profile can enhance collective organizational engagement. In the “High (L)-Low (E)” profile, participative leadership, by providing intrinsic motivation, will constantly shift employees’ LMX perceptions of the employee-leader relationships and develop towards high-quality LMX congruence. It indicates that the interaction between participative leadership and the “High (L)-Low (E)” can enhance collective organizational engagement.

Thus, the following hypotheses are developed.

*H5:* The interaction between participative leadership and LMX incongruent profiles is positively associated with collective organizational engagement.

*H5a:* The interaction between participative leadership and “High (L)-Low (E)” profile is positively associated with collective organizational engagement.

*H5b:* The interaction between participative leadership and the “Low (L)-High (E)” profile is positively associated with collective organizational engagement.

## 2. Method

This study adopts a cross-sectional matched survey design. Therefore, the statistical analyses (including polynomial regression and response surface analysis) examine predictive associations among variables rather than causal association. Interpretations of the findings are limited to relational and predictive patterns. This study adopted a cross-sectional, multi-source matched survey design. Data were collected from leader–employee dyads at the team level to examine predictive associations among participative leadership, leader–member exchange (LMX) congruence, and collective organizational engagement.

A matched survey approach was used to reduce single-source bias and enhance the accuracy of relational measures. The cross-sectional design was appropriate for examining associative patterns among variables within ongoing organizational contexts, although causal inferences cannot be drawn.

### 2.1 Measures

#### (1) Participative leadership.

The Huang, Iun, & Liu’s (2010) [48] adaptation of Arnold, Arad, & Rhoades’ (2000) [49] scale was used to measure participative leadership. The adapted scale consists of the following 6 items. My leader encourages me to voice my ideas; my leader willingly listens to my ideas; my leader accepts my rationalization proposals; my leader provides me with the opportunity to make proposals; my leader will not directly and rashly reject my proposals; my leader will not act in disregard of our proposals. Miao (2013, 2014) has proved the reliability and validity of this scale in Chinese contexts [12,23].

#### (2) LMX.

The 7-item scale of Graen & Uhl-Bien’s (1995^)^ [50] was used to measure the LMX rating of employees. This LMX scale for employees’ ratings mainly consists of 7 items (such as my perception of the leader’s expectation, and the degree to which my leader knows about my problems and needs at work). The LMX scale for leaders’ ratings was adapted from the statements of Tsui, Pearce, & Porter (1997) [51] and Matta, Scott, & Koopman (2015) [20] by changing the employee standpoint into the leader standpoint. This scale also consists of 7 items. For example, my subordinates generally know how to meet my expectations, and I know well about the problems and needs of my subordinates at work.

#### (3) Collective organizational engagement.

Consistent with the referent-shift approach, respondents evaluated engagement at the team level rather than reporting their own individual engagement. Collective organizational engagement was measured using the scale of Rich et al. (2010) [52]. The referent-shift method of Barrick et al. (2015) [9] was used so that employees could rate from at the organizational level. Specifically, in the instructions and item statements, employees are required to evaluate at the organizational or team level. For example, our members will fully engage in work, and I find that almost all my colleagues are working actively and diligently.

Since leaders and their employees should be investigated separately in this research, the leader-employee matching survey method was adopted for data collection. Therefore, the research primarily used two questionnaires. One questionnaire was filled out by leaders, comprising 7 items. And the other one was filled out by employees, consisting of 19 items. In addition, as differences in age, gender, working age, and other individual variables may affect the perceptions of participative leadership, LMX, and collective organizational engagement, this research used age, gender, education level, working, and enterprise type as control variables. Hence, the total scale for leaders has 12 items, while the total scale for employees has 24 items.

#### 2.2 Data collection.

##### (1) Procedures

This research primarily relied on two channels (two projects of our research group and our social networks) to distribute and collect questionnaires. In the first-part (project-based) data collection, human resources of the enterprises served by the projects were utilized, with 3–5 teams selected from each enterprise for leadership and employee questionnaire surveys. To ensure data accuracy, one-to-one matching between leaders and employees was applied for questionnaire distribution and collection. Data was collected according to the following steps. Firstly, employees within the teams were assigned and recorded with identification (ID) numbers, and the leader and employee questionnaires were marked with corresponding numbers. Secondly, questionnaires were distributed to each employee in the team. Moreover, questionnaires were distributed to the leaders according to the recorded ID numbers, and leaders were asked to rate employees based on their recorded ID numbers. Lastly, the leader-employee matching questionnaires were collected and placed in files for data analysis. The distribution and collection of questionnaires through social networks also followed the above procedures

#### (2) Research samples.

This paper primarily adopted a matching method for data collection to acquire samples from two main sources. The first part of the data was collected from two government-supported manufacturing transformation and management upgrading initiatives in which members of the research team were involved as external consultants. These initiatives aimed to improve organizational processes, digital systems, and managerial practices within participating firms. The participating firms served as the study setting during the implementation phase of these initiatives. In the project implementation, 168 questionnaires were distributed to 35 teams and all of them were returned. Out of the 168 questionnaires, 117 matching questionnaires from 26 teams were valid, so the effective response rate was 69.6%. The second-part sample data mainly came from corporate organizations in Hubei, Shandong, and Liaoning provinces, covering industries such as services, information technology, real estate, and fast-moving consumer goods. A total of 202 questionnaires were distributed to 42 teams, with 178 returned. Out of the 178 questionnaires, 126 of them were valid, indicating an effective response rate of 62.4%. In summary, a total of 242 valid matching questionnaires were available in this research. Therefore, the study setting consisted of commercial firms undergoing organizational upgrading or management transformation processes, providing a meaningful context for examining leadership behavior and collective engagement dynamics.

The 54 commercial firms involved in this study operated across multiple industries, including manufacturing, information technology, services, real estate, and fast-moving consumer goods. Although they differed in industry sector and ownership structure (state-owned, private, foreign-funded, and joint ventures), most were medium-sized organizations undergoing internal restructuring or management upgrading initiatives during the data collection period. This shared transitional context required frequent leader–employee interaction and coordination, making leadership behavior and leader–member relational alignment particularly salient. To reduce potential contextual confounding association, enterprise type was included as a control variable in subsequent analyses.

#### (3) Control of potential bias.

The study implemented several procedural and statistical remedies to mitigate potential sources of bias. First, to reduce common method bias, data were collected from multiple sources using a matched leader–employee design. Participative leadership and employee-rated LMX were reported by employees, whereas leader-rated LMX was reported by leaders, thereby reducing single-source variance. In addition, Harman’s single-factor test was conducted, and the first unrotated factor did not account for the majority of variance, suggesting that common method bias was unlikely to substantially distort the results. Second, to address potential confounding bias, demographic variables (gender, age, education, and working age) and enterprise type were included as control variables in all regression models. Third, to reduce artificial classification bias in LMX profiles, latent profile analysis (LPA) was employed to validate naturally occurring congruence configurations rather than relying solely on median-split procedures. Finally, multicollinearity diagnostics were examined prior to regression analyses, and no severe multicollinearity issues were detected.

#### (4) Descriptive statistics of the samples.

For the descriptive statistics, among the 243 samples, 124 were male and 119 were female, accounting for 51.0% and 49.0%, respectively. In terms of age, 23 of them aged 20 or below, accounting for 9.5% of the total; 169 of them aged 20 to 30, accounting for 69.5%; 47 of them aged 30 to 40, accounting for 19.3%; 4 of them aged 40 to 50, accounting for 1.6%. In terms of the education level, 12 of them received a high school education, accounting for 4.9% of the total; 21 of them received a college education, accounting for 8.6%; 109 of them had a bachelor's degree, accounting for 44.9%; 101 of them had a master's degree or higher. In terms of the working age, 152 of them had worked for 1–3 years, accounting for 62.1% of the total; 47 of them had worked for 3–5 years, accounting for 19.3%; 36 of them had worked for 5–10 years, accounting for 14.8%; 9 of them had worked for 10 years or longer, accounting for 3.7%. In terms of the enterprise type, 91 of them came from state-owned enterprises, accounting for 37.4%; 68 of them came from private enterprises, accounting for 28.0%; 47 of them came from foreign-funded enterprises, accounting for 19.3%; 34 of them came from Sino-foreign joint ventures, accounting for 14.0%.

## 3. Results

### 3.1 Data analysis

#### (1) Data aggregation test.

A team-level data aggregation test was conducted using the indexes: the within-team interrater congruence coefficient R, the intra-group correlation coefficient ICC(1), and the inter-group correlation coefficient ICC(2). The mean R, median R, ICC(1), and ICC(2) values for each variable were listed as follows: Participative leadership (0.82, 0.85, 0.45, 0.93), LMX (0.87, 0.88, 0.47, 0.92), collective organizational engagement (0.89, 0.90, 0.49, 0.95). The median and mean R-values of all three variables were greater than the recommended threshold of 0.7, and their ICC(1) and ICC(2) values exceeded the critical values of 0.12 and 0.7, respectively. Therefore, it is feasible to aggregate and average the individual-level data at the team level.

#### (2) Reliability test.

SPSS 20.0 was applied for data reliability tests, primarily including reliability analysis and item analysis of the variables. The reliability test results for the variables are shown in Table 2 below. The item analysis was conducted mainly according to two criteria: (1) the Cronbach’s α coefficient of a deleted item being greater than the overall Cronbach’s α coefficient of the variable, and (2) the Corrected Item-Total Correlation (CITC) value being greater than 0.4. The test results show that all scales used in this research have α coefficients greater than 0.7 and pass the reliability tests, indicating high internal congruence.

**Table 2:** Reliability Test Results of the Scales (N = 243).

Variable	Cronbach’s α coefficient
PL	0.941
ELMX	0.931
LLMX	0.860
COE	0.912

**Note**: In each model, “PL” denotes Participative Leadership; “ELMX” denotes Employee LMX; “LLMX” denotes Leader LMX and “COE” denotes Collective Organizational Engagement.

### (3) Validity test

This paper tested the validity of the measurement scales mainly from two perspectives. All scales used were well-established and previously validated in domestic and international literature, and they all passed a pre-test, indicating good content validity. Moreover, a confirmatory factor analysis (CFA) was conducted using Amos 19.0 to validate the constructs of participative leadership, employee LMX, leader LMX, and collective organizational engagement. The validity analysis results are shown in Table 3.

**Table 3:** Validity Test Results of the Scales（N = 243）.

Model	χ^2^/df	GFI	NFI	IFI	CFI	RMSEA
Four-factor model: PL，ELMX，LLMX，COE	2.034	0.912	0.907	0.911	0.930	0.036
Three-factor model: PL+ ELMX，LLMX，COE	3.982	0.889	0.865	0.891	0.893	0.051
Two-factor model: PL+ ELMX+ LLMX，COE	4.181	0.874	0.832	0.861	0.873	0.127
Single-factor model: PL+ ELMX+ LLMX+ COE	7.433	0.861	0.817	0.813	0.789	0.312

According to the above analysis, only the four-factor model yields fit indices within an acceptable value range and has the best model fit.

The composite reliability (CR) values ranged from 0.911 to 0.927, exceeding the recommended threshold of 0.70. The average variance extracted (AVE) values ranged from 0.625 to 0.644, all above the recommended cutoff of 0.50, indicating satisfactory convergent validity. (See Table 4).

**Table 4:** Standardized Factor Loadings, Composite Reliability, and AVE.

Construct	Item	Standardized Loading	CR	AVE
Participative Leadership	PL1	0.71	0.91	0.63
	PL2	0.89		
	PL3	0.73		
	PL4	0.82		
	PL5	0.79		
	PL6	0.81		
Employee LMX	ELMX1	0.72	0.92	0.63
	ELMX2	0.79		
	ELMX3	0.81		
	ELMX4	0.82		
	ELMX5	0.74		
	ELMX6	0.75		
	ELMX7	0.89		
Leader LMX	LLMX1	0.84	0.93	0.64
	LLMX2	0.84		
	LLMX3	0.82		
	LLMX4	0.79		
	LLMX5	0.77		
	LLMX6	0.73		
	LLMX7	0.82		
Collective Organizational Engagement	COE1	0.79	0.91	0.67
	COE2	0.78		
	COE3	0.81		
	COE4	0.87		
	COE5	0.83		

#### (4) Common method variance test.

The Harman’s single-factor test was performed to check the common method variance in the measurement data. The explained variance of the rotated first principal component was 29.391%, while the total explained variance was 85.362%. No single factor could explain a large portion of the variance, indicating that common method variance was small in this research.

#### (5) Latent profile analysis.

Previous research generally used the median-split method to classify leaders’ and subordinates’ LMX perceptions into high/low levels, thus creating four profiles. The sample data were classified accordingly, as shown in Table 5.

**Table 5:** Sample distribution of four LMX profiles (N = 243).

profile	Sample size	Percentage
“High(L)-High(E)” profile	60	24.69%
“Low(L)-Low(E)” profile	45	18.52%
“High(L)-Low(E)” profile	63	25.93%
“Low(L)-High(E)” profile	75	30.86%

However, some scholars argue that such artificial classification will distort the continuous distribution of variables and consequently cause information loss. To more scientifically identify the natural profiles of the leaders’ and employees’ LMX ratings, Latent Profile Analysis (LPA) was used to verify the four LMX profiles. LPA was applied to build latent profile models by taking the mean scores of two continuous variables (LMX perceptions of 243 employees and leaders) across items as observed variables. The model fit indices for different numbers of profiles are analyzed, as shown in Table 6.

**Table 6:** Comparison of model fit indices in latent profile analysis (N = 243).

Number of profiles	LL	f	AIC	BIC	SSA-BIC	LMR (p-value)	BLRT (p-value)	Entropy
1	−620.18	4	1248.36	1262.54	1251.23	—	—	—
2	−554.72	7	1123.44	1145.37	1129.45	<0.001	<0.001	0.851
3	−518.06	10	1056.12	1085.80	1065.27	0.012	<0.001	0.892
4	−495.13	13	1016.27	1053.70	1028.56	0.038	<0.001	0.915
5	−489.94	16	1011.88	1057.06	1027.31	0.215	0.105	0.901

As shown in Table 5, the 4-profile model is the best-fitting one as it has the smallest AIC, BIC, and SSA-BIC values among all models. Moreover, when the number of profiles increases from 3 to 4, the Lo-Mendell-Rubin (LMR) test is significant (p = 0.038 < 0.05), and the Entropy value reaches its highest (0.915), indicating good classification. When the number increases from 4 to 5, the LMR test is insignificant (p = 0.215 > 0.05), and the BIC and SSA-BIC values increase, indicating a decline in model fit, so the 5-profile model is rejected. Therefore, the 4-profile model is adopted as the optimal solution. Such classifications align with practical observations and prior research findings.

### 3.2 Hypothesis tests

#### (1) Correlation analysis.

Correlation analysis was conducted as a preliminary diagnostic step to examine bivariate associations among variables and assess potential multicollinearity prior to polynomial regression and response surface analysis. The purpose was not to infer causal association, but to ensure the suitability of the data structure for subsequent regression modeling. The mean values, standard deviations (SD), and correlation coefficients of the variables in this research are shown in Table 7 below.

**Table 7:** Correlation Analysis (N = 243).

Variable	Mean	SD	1	2	3	4	5	6	7	8
1 Gender	1.49	0.50	1							
2 Age	2.19	0.51	−.049	1						
3 Education	3.23	0.81	.016	.083	1					
4 Working age	1.60	0.87	−.080	.035	.008	1				
5 LP	3.23	1.04	−.190^**^	.043	−.107	−.014	1			
6 ELMX	3.50	0.81	−.220^**^	−.006	.018	.080	.610^*^	1		
7 LLMX	3.56	0.84	.062	.102	−.034	−.021	.068	−.109	1	
8 COE	3.14	0.91	−.257^**^	.083	.045	.151^*^	.428^**^	.619^**^	−.101	1

**Note**: ** indicates significance at p < 0.01; * indicates significance at p < 0.05.

According to a preliminary correlation analysis, participative leadership (PL) has a significant positive effect on employee LMX (ELMX) and collective organizational engagement (COE), while employee LMX has a positive effect on collective organizational engagement. Variables show correlations, facilitating the subsequent regression analyses.

#### (2) Effect of participative leadership on collective organizational engagement.

SPSS 20.0 was used to test the association of participative leadership on collective organizational engagement. The test results are shown in Table 8 below.

**Table 8:** Regression analysis results of participative leadership on collective organizational engagement (N = 243).

	Dependent variables			
	Model 1		Model 2	
**Control variable**	Correlation coefficient	t-value	Correlation coefficient	t-value
Gender	−.407***	−3.921	−.279***	−2.896
Age	.137	1.329	.108	1.150
Education	.503	.670	.088	1.494
Working age	.040*	2.066	.134**	2.477
**Independent variables**				
Participative leadership	–	–	.326***	6.988
F	6.042***		15.601***	
R^2^	0.092		.248	
Adjusted R^2^	0.077		.232	

Note: *** indicates significance at the level of p < 0.001; ** indicates significance at p < 0.01; * indicates significance at p < 0.05.

As shown in Table 7, Participative leadership significantly predicts collective organizational engagement (β = 0.326, p < 0.001), supporting Hypothesis 1.

#### (3) Effect of LMX on collective organizational engagement.

Hierarchical regression analysis was performed using SPSS20.0 to check the effect of LMX congruence, LMX incongruence, and the four LMX profiles on collective organizational engagement. The regression analysis results are shown in Table 9 below.

**Table 9:** Regression analysis results of LMX on collective organizational engagement (N = 243).

Variable	Dependent variable					
	Model 1	Model 2	Model 3	Model 4	Model 5	Model 6
**Control variable**						
Gender	−.038*	−.029	−.744	.246	−.492	.264
Age	−.081	.056	−.119	.382	.177	.189
Education	.167*	.111	.168	.363	.016	.233
Working age	.102	.109	.051	.010	.107	−.056
**Independent variable**						
LMX congruence	.098***					
LMX incongruence		.171***				
“High (L)-Low(E)”			.078*			
“Low(L)-Low(E)				.135*		
“High(L)-Low(E)”					.021	
“Low (L)-High(E)”						.107**
F	9.655***	7.756***	5.592***	4.461***	3.378*	3.516**
R^2^	.328	.227	.341	.364	.229	.203
Adjusted R^2^	.294	.198	.280	.282	.161	.145

**Note**: *** indicates significance at p < 0.001; ** indicates significance at p < 0.01; *denotes significance at p < 0.05.

As shown in Table 8, both LMX congruence and incongruence profiles have a positive effect on collective organizational engagement (β = 0.098, p < 0.001; β = 0.171, p < 0.001). However, LMX congruence has two profiles, and LMX incongruence equally has two profiles, so it is unreasonable to conclude the effect of LMX on collective organizational engagement solely based on LMX congruence and incongruence. By investigating the association of the “High (L)-High (E)” profile and the “Low (L)-Low (E)” profile on collective organizational engagement, we conclude that both the two congruence profiles had a significant positive impact on collective organizational engagement (β = 0.078, p < 0.05; β = 0.135, p < 0.05), supporting Hypothesis 2a and Hypothesis 2b in this research.

In LMX congruence profiles, the positive effect of the “high (L)-high (E)” profile on collective organizational engagement aligns with the traditional matching perspective. Specifically, when both leaders and employees within an organization perceive high LMX ratings, leaders will provide more support to employees while employees will willingly accept the leaders’ support and actively engage in their work. Unexpectedly, “Low (L)-Low (E)” will not weaken collective organizational engagement. In the “Low (L)-Low (E)” profile, leaders and employees perceive their exchanges as transactional, support provided by leaders can meet employees’ expectations, and employees can complete their work well. According to Role theory, when the traits and behaviors of leaders and employees at work can meet each party’s expectations, it will achieve balance in their relationship and improve their work performance [36]. The findings of this research further verify the above-mentioned theory.

To further investigate the differences in the association of LMX congruence, LMX incongruence, and the four LMX profiles on collective organizational engagement, this paper adopted the methods of polynomial regression and response surface analysis to verify the research hypotheses.

**Fig 1:**
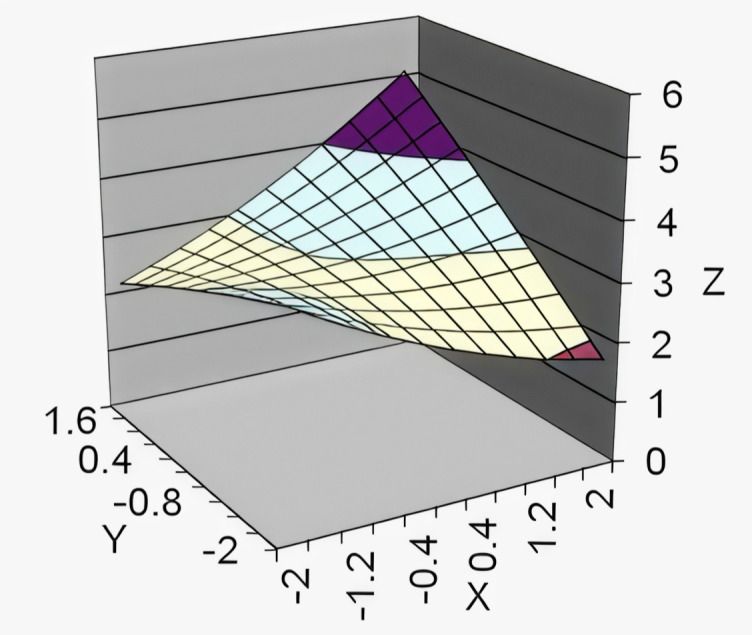
Response surface analysis of the effect of LMX congruence and incongruence on collective organizational engagement.

According to a comprehensive analysis of Table 9 and Fig 1, when the response surface lies along the congruence line (L_f_ = L_e_), the curvature is significant and positive (curvature = 0.299, p < .05), indicating that a higher congruence level between the leader's and subordinate's perceptions of LMX had a greater effect on collective organizational engagement. Moreover, the slope has a significantly positive value (slope = 0.349, p < .01), suggesting that the “High (L)-High (E)” profile has a greater effect on collective organizational engagement than the “Low (L)-Low (E)” profile. Hence, Hypothesis 2c is supported.

In LMX incongruence profiles, the “Low (L)-High (E)” profile shows a significantly positive effect on collective organizational engagement (β = 0.017, p < .01), thus verifying Hypothesis 3b. In contrast, the “High (L)-Low (E)” profile shows no significant correlation with collective organizational engagement (β = 0.021, ns), thus rejecting Hypothesis 3a. It is perhaps because when employees perceive their relationship with leaders as transactional, the leaders’ encouragement and non-material incentives may have little effect on these employees who expect more material incentives.

When the response surface lies along the incongruence line (L_f_ = -L_e_), the slope is significant and negative. It indicates that the “Low (L)-High (E)” profile has a more significant effect on collective organizational engagement than the “High (L)-Low (E)” profile, thus supporting Hypothesis 3c. Previous matching research suggests that employees play a more critical role than leaders in the workplace [46], which aligns with the research findings in this paper. However, although the “Low (L)-High (E)” profile has a positive effect on enhancing collective organizational engagement, its effect is smaller than that of the “Low (L)-Low (E)” profile. For example, the point at coordinates (−2, 1) in the figure shows a smaller effect on collective organizational engagement than the point at (−2, −2). In the “Low (L)-High (E)” profile, employees perceive a high LMX rating, which is socio-emotional according to Matta et al. (2015), and has some association on enhancing employee work engagement. However, leaders perceive LMX as transactional and may go against employees’ emotional expectations. Both leaders and employees perceive LMX as transactional in the “Low (L)-Low (E)” profile, so emotional expectations are fulfilled. This indicates that higher congruence in LMX ratings can more effectively enhance collective organizational engagement.

#### (4) Association of the interaction between LMX and participative leadership on collective organizational engagement.

Furthermore, this paper adopted the research ideas of Edwards and Cable (2009) to multiply the leaders’ and employees’ LMX ratings by their respective regression coefficients and sum them to create a composite variable [53]. The specific analysis results are shown in Table 11 below.

**Table 11:** Test results of the effect of the matching between LMX and participative leadership on collective organizational engagement (N = 243).

	Dependent variable					
	Model 1	Model 2	Model 3	Model 4	Model 5	Model 6
**Control variable**						
Gender	−.360**	−.024	−.644***	.305	−.526**	.380**
Age	−.071	.119	−.116	.373*	.199	.213
Education	.157	.092	.210	.384***	.002	.249**
Working age	.128	.099	.006	.021	.105	−.022
**Independent variable**						
LMX congruence*PL	.016***					
LMX incongruence*PL		.030***				
“H(L)-H(E)” profile*PL			.019**			
“L(L)-L(E)” profile*PL				−.024*		
“H(L)-L(E)” profile*PL					−.003	
“L(L)-H(E)” profile*PL						.024***
F	9.239***	13.376***	5.592***	4.054**	3.343**	4.607**
R^2^	.318	.336	.341	.324	.227	.250
Ajudsted R^2^	.284	.311	.280	.258	.159	.196

**Note**: *** denotes significance at the 0.001 level; ** denotes significance at the 0.01 level; * denotes significance at the 0.05 level; “PL” denotes participative leadership

According to the analysis results in Table 10, when LMX congruence and incongruence profiles interact with participative leadership, both of them show a significantly positive effect on collective organizational engagement (β = 0.016, p < 0.001; β = 0.030, p < 0.001). However, since LMX congruence has two profiles and LMX incongruence equally has two profiles, this paper further analyzed the association of the interactions between the four LMX profiles and participative leadership on collective organizational engagement. According to the analysis results, the interaction between the “High(L)-High(E)” profile and participative leadership is positively associated with collective organizational engagement (β = 0.019, p < 0.01); the interaction between the “L(L)-L(E)” profile and participative leadership has a significantly negative effect on collective organizational engagement (β = −0.024, p < 0.05); the interaction between the “High(L)-L(E)” profile and participative leadership has no significant effect on collective organizational engagement (β = −0.003, p > 0.01); the interaction between the “L(L)-High(E)” profile and participative leadership is positively associated with collective organizational engagement (β = 0.024, p < 0.001). These findings support H4a and H5b and reject H4b and H5a.

**Table 10:** Polynomial regression and response surface analysis results.

Variable	Dependent variable		
	Model 1	Model 2	Model 3
**Control variable**			
Gender	−.412***	−.190*	−.036
Age	.018	.091	−.026
Education	.050	.033	.171
Work age	.125*	.085	.102
**Independent variable**			
Leader LMX rating（X）b_1_		.202	.132
Employee LMX rating（Y）b_2_		.164**	.217*
Square of X（X^2^）b_3_			.060
X*Y（X*Y）b_4_			.257*
Square of Y（Y^2^）b_5_			−.018
Adjusted R^2^	0.070	0.402	0.477
**Response Surface Analysis**			
Critical point	（−0.74 −0.14）		
Principal Axis 1	y = 0.43x + 0.77		
Principal Axis 2	y = −1.10x-1.29		
congruence L_f_ = L_e_			
Slope（b_1_ + b_2_）	.349**		
Curvature（b_3_ + b_4_ + b_5_）	.299*		
Incongruence L_f_ = -L_e_			
Slope（b_1_-b_2_）	−.085**		
Curvature（b_3_-b_4_ + b_5_）	−.215		

Note: ***denotes significance at p < 0.001; ** denotes significance at p < 0.01; *denotes significance at p < 0.05.

Notably, Table 10 shows that the interaction between “L(L)-L(E)” profile and participative leadership has a negative effect on collective organizational engagement—a finding contrary to the initial hypothesis H4b.This unexpected finding can be explained as follows. In the “Low(L)-Low(E)” leader-employee exchanges, both leaders and employees have transactional expectations. However, when leaders employ resource-exchange methods with transactional intentions and give employees excessive encouragement, participation rights, and decision-making authority, employees may perceive it as “superficial” and feel “overwhelmed” [54] and thus feel disappointed. According to the social exchange rule, employees will generate negative feelings and reduce their engagement for achieving psychological equilibrium [36].

H5a is rejected possibly for the following reasons. In the “High(L)-Low(E)” profile, employees perceive their exchanges with leaders as transactional, so intangible incentives from leaders may produce limited effect. When participative leadership motivates employees to engage in decision-making and problem-solving activities, the motivation will has little effect without providing obvious material motivation. So it is easy to understand why the interaction between the “High(L)-Low(E)” and participative leadership shows no significant effect on collective organizational engagement.

## 4. Discussion

First, the positive association between participative leadership and collective organizational engagement is broadly consistent with prior studies that have linked participative or empowering leadership to individual-level engagement and performance outcomes. However, while earlier research has primarily focused on individual employee engagement, the current study extends this line of inquiry by examining engagement at the collective level. By demonstrating that participative leadership is associated with shared engagement perceptions within teams, the study provides additional support for the multilevel implications of participative leadership.

Second, the findings regarding LMX congruence align with prior work emphasizing the importance of perceptual agreement in leader–member relationships. Earlier research has shown that alignment in LMX perceptions is associated with favorable individual outcomes, such as job satisfaction and organizational citizenship behavior. The present study extends these insights by showing that congruence patterns are differentially associated with collective organizational engagement, suggesting that perceptual alignment has implications beyond dyadic exchanges and may influence emergent team-level states.

At the same time, the profile-specific results refine existing assumptions in the LMX literature. While much prior research implicitly assumes that high-quality LMX is uniformly beneficial, the present findings indicate that relational alignment and employees’ perceptions play a particularly salient role. Incongruent configurations, especially those in which employees report higher LMX perceptions than leaders, exhibit distinctive association patterns. This nuance contributes to a more differentiated understanding of LMX dynamics.

Finally, the interaction patterns observed between participative leadership and LMX profiles resonate with contingency perspectives in leadership research. Previous studies have suggested that the effectiveness of leadership behaviors depends on contextual and relational factors. The present findings provide empirical evidence that relational congruence functions as an important boundary condition, shaping how participative leadership relates to collective engagement outcomes.

### 4.1 Theoretical implications

First, this research integrates intrinsic motivation theory, role theory, and social contagion theory into a unified multilevel mechanism to explain the emergence of collective organizational engagement. Rather than treating these theories independently, this study clarifies how participative leadership activates intrinsic motivation, how LMX congruence ensures role alignment, and how social contagion transforms individual psychological states into collective engagement. This integration advances engagement research by offering a coherent theoretical explanation of collective-level emergence.

Moreover, this study advances the understanding of LMX by emphasizing the dyadic nature of LMX congruence. The findings indicate that the associations between participative leadership, LMX congruence, and collective organizational engagement are conditional rather than uniformly positive. While prior LMX research has typically adopted a single-source perspective—either the leader’s or the employee’s perception—this study highlights the importance of perceptual alignment between leaders and employees. By conceptualizing LMX congruence as a profile-based construct and employing polynomial regression and response surface analysis, this study systematically examines the differential associations of congruent and incongruent LMX perceptions with collective organizational engagement, thereby enriching the dyadic interaction perspective in LMX research.

Furthermore, this study sheds light on the interplay between leadership style and relational alignment. By integrating participative leadership and LMX congruence profiles within a unified analytical framework, it demonstrates that the association between participative leadership and collective organizational engagement depends on specific LMX configurations. Participative leadership is not uniformly associated with higher levels of collective engagement across all relational contexts; rather, its association varies according to the degree of perceptual congruence between leaders and employees. These findings extend contingency leadership perspectives by underscoring the importance of relational alignment as a boundary condition in understanding how leadership relates to collective-level outcomes.

### 4.2 Practical implications

First, organizations may benefit from fostering participative leadership practices that encourage employee involvement in decision-making and problem-solving processes. By granting employees greater voice and autonomy, leaders can create conditions that are conducive to higher levels of collective engagement. Participative leadership may be particularly valuable in environments that require coordination, shared responsibility, and collective effort.

Second, the results highlight the importance of monitoring and managing leader–member exchange (LMX) alignment. Rather than focusing solely on leaders’ evaluations of relationships, organizations should pay close attention to employees’ perceptions of LMX quality. The degree of perceptual congruence between leaders and employees appears to be associated with different patterns of collective engagement. Regular climate surveys or structured feedback mechanisms can be used to assess relational alignment and identify discrepancies between leader and employee perceptions.

Third, the findings suggest that relational context matters. When leader–employee relationships are perceived as socio-emotional by both parties, non-material incentives such as trust, recognition, and developmental support may be particularly meaningful. In contrast, when relationships are perceived as more transactional, tangible rewards and clear performance expectations may play a more prominent role. Accordingly, leadership practices may be more effective when they are aligned with employees’ relational expectations.

Finally, from a contingency perspective, no single leadership approach is universally associated with higher collective engagement across all relational configurations. Leaders should therefore adopt a context-sensitive approach, adjusting their leadership behaviors based on the quality and alignment of leader–employee relationships. Developing awareness of relational dynamics and fostering open communication can help reduce perceptual discrepancies and strengthen collective engagement within teams.

### 4.3 Research limitations and future research directions

First, although multiple procedural remedies were implemented to mitigate common method variance (CMV), including multi-source matched data and Harman’s single-factor test, the latter approach has limited sensitivity. Future research may adopt more rigorous techniques, such as marker variables or latent method factor modeling, to better assess and control for potential CMV.

Second, the study was conducted within a Chinese organizational context, where relational norms such as guanxi may shape how leaders and employees interpret socio-emotional and transactional exchanges. The profile-specific associations observed—particularly the relatively stronger positive association of the “Low (L)-High (E)” configuration—may reflect culturally embedded relational expectations. Future studies could examine these patterns in different cultural contexts or incorporate cultural dimensions (e.g., collectivism, relationship orientation) as moderating variables to identify potential boundary conditions.

Third, the cross-sectional design limits the ability to establish temporal ordering among variables. Although polynomial regression and response surface analysis enable the examination of complex associative patterns, causal inferences cannot be drawn. Longitudinal designs, experimental approaches, or field interventions would help clarify developmental processes and strengthen causal interpretation.

Fourth, some regression coefficients were statistically significant but relatively small in magnitude. While this is common in profile-based and congruence research, future research should examine the practical significance of these associations and explore whether the observed patterns replicate across larger and more diverse samples.

Fifth, the negative association observed between participative leadership and collective engagement under the “Low (L)-Low (E)” profile warrants further investigation. Future studies may explore underlying mechanisms, such as psychological contract breach, role overload, or expectancy misalignment, to better understand why participative behaviors may not align with transactional relational expectations in certain contexts.

Finally, LMX perceptions are likely to evolve over time. The present design does not capture dynamic shifts in leader–employee alignment. Future research may employ experience sampling methods or longitudinal tracking designs to examine how LMX congruence develops and how changes in relational alignment relate to collective engagement over time.

## 5 Conclusion

In this paper, 243 leader-employee questionnaires were collected and analyzed to reach the following conclusions. Participative leadership is positively associated with collective organizational engagement; the association of LMX congruence on collective organizational engagement vary by profile; in the case of LMX congruence, the “High (L)-High (E)” has a greater positive effect on collective organizational engagement than the “Low (L)-Low (E)” profile, while in the case of LMX incongruence, the “Low (L)-High (E)” profile has a greater positive effect on collective organizational engagement than the “High (L)-Low (E)” profile; The interaction association between participative leadership and LMX profiles show partial support for our hypotheses. Specifically, significant positive association were observed in the “High (L)-High (E)” and “Low (L)-High (E)” profiles, whereas the association were non-significant or negative in other configurations.
